# Nutrigerontology: a key for achieving successful ageing and longevity

**DOI:** 10.1186/s12979-016-0071-2

**Published:** 2016-05-21

**Authors:** Anna Aiello, Giulia Accardi, Giuseppina Candore, Giuseppe Carruba, Sergio Davinelli, Giuseppe Passarino, Giovanni Scapagnini, Sonya Vasto, Calogero Caruso

**Affiliations:** Department of Pathobiology and Medical Biotechnologies, University of Palermo, Palermo, Italy; Division of Research and Internationalization, ARNAS-Civico Di Cristina e Benfratelli, Palermo, Italy; Department of Medicine and Health Sciences, School of Medicine, University of Molise, Campobasso, 86100 Italy; Department of Biology, Ecology and Earth Science, University of Calabria, Rende (CS), 87036 Italy; Department of Biological Chemical and Pharmaceutical Sciences and Technologies (STEBICEF), University of Palermo, Palermo, Italy and Institute of biomedicine and molecular immunology “Alberto Monroy” CNR, Palermo, Italy

**Keywords:** Ageing, Longevity, Mediterranean Diet, Nutraceuticals, Nutrigerontology, Phytochemicals

## Abstract

During the last two centuries the average lifespan has increased at a rate of approximately 3 months/year in both sexes, hence oldest old people are becoming the population with the fastest growth in Western World. Although the average life expectancy is increasing dramatically, the healthy lifespan is not going at the same pace. This underscores the importance of studies on the prevention of age-related diseases, in order to satisfactorily decrease the medical, economic and social problems associated to advancing age, related to an increased number of individuals not autonomous and affected by invalidating pathologies. In particular, data from experimental studies in model organisms have consistently shown that nutrient signalling pathways are involved in longevity, affecting the prevalence of age-related loss of function, including age-related diseases. Accordingly, nutrigerontology is defined as the scientific discipline that studies the impact of nutrients, foods, macronutrient ratios, and diets on lifespan, ageing process, and age-related diseases. To discuss the potential relevance of this new science in the attainment of successful ageing and longevity, three original studies performed in Sicily with local foods and two reviews have been assembled in this series. Data clearly demonstrate the positive effects of nutraceuticals, functional foods and Mediterranean Diet on several biological parameters. In fact, they could represent a prevention for many age-related diseases, and, although not a solution for this social plague, at least a remedy to alleviate it. Thus, the possibility to create a dietary pattern, based on the combined strategy of the use of both nutraceuticals and functional foods should permit to create a new therapeutic strategy, based not only on a specific bioactive molecule or on a specific food but on a integrated approach that, starting from the local dietary habits, can be led to a “nutrafunctional diet” applicable worldwide.

## Background

Nutrigerontology is defined as the scientific discipline that studies the impact of nutrients, foods, macronutrient ratios, and diets on lifespan, ageing process, and age-related diseases. Its goal is to investigate about compounds, foods, and diets that can reduce the risk of ageing-related diseases and increase the healthy lifespan, so achieving successful ageing and longevity [1].

Many definitions of longevity and successful ageing have been proposed but none has been accepted yet. In May 2012, a group of scientists and clinicians met in Athens (Greece) to consider the relevance of ageing, longevity, exceptional longevity and related genetic and non genetic markers. The workshop led to the creation of a consensus statement to highlight the importance of a common view related to these processes, since they represent phenotypes that are rapidly spreading worldwide [2].

As reported in this panel: “Successful ageing involves avoidance (or late onset) of age-related diseases including cardiovascular disease which is the main cause of death, and other organ specific diseases, disability, preservation of desirable cognitive and physical function and social activities throughout the lifespan”. Moreover, it proposes the definition of exceptional longevity in relative and absolute terms, on the basis of demographic data. It quotes that: ““Relative” suggests that longevity is concept country/population specific and must take into consideration the life expectancy of the different populations/countries, which show great variability owing to historical, anthropological and socio-economic differences. In “absolute” terms longevity could be defined according to the maximum lifespan attained and scientifically validated by human beings in the planet” [2].

Also, the statement reported the main genes related to longevity and ageing. Such genes and their encoded proteins are included in a variety of signalling pathways, i.e., the nutrient sensing insulin/insulin-like growth factor-1 (IGF-1) or mammalian target of rapamicin (mTOR), the oxidative stress and anti-oxidant ones and that involved in the control of immune-inflammatory responses [2].

In addition, it is becoming clear that epigenetic changes linked to environmental/life style factors (such as physical activity, diet and emotional stress) play a role in longevity attainment [2, 3].

In particular, a nutrient-sensing pathway is activated by nutrients that trigger signals that lead to a downstream activation of genes involved in ageing processes. The complex relationship among nutrition and healthy ageing is not fully understood, but evidences have already demonstrated that both in animal models and humans, dietary intervention can positively modulate ageing process, preventing or decreasing various age-related diseases and their generalized pro-inflammatory status, the inflammageing [4, 5]. So, healthy diets that not overstimulate mTOR and insulin/IGF-1 pathways because poor in refined sugars and animal proteins can substantially reduce the risk of age-related diseases, hence favouring successful ageing and longevity [6, 7]. On the contrary, a bad dietary lifestyle accelerates the ageing process, activating the pathways with nutrients, growth factors and mitogenic stimuli, so accelerating ageing phenotype [1].

Mediterranean Diet (MedDiet) is one of the most studied healthy dietary patterns. It is an alimentary regimen with low-glycaemic index and low animal protein intake that contains phytochemical compounds found in vegetables, fruits, red wine, olive oil or nuts, with anti-inflammatory and anti-oxidant effects. This pattern, that more than a diet could be defined a lifestyle, owes its health properties also to the nutraceuticals [1, 8–11], defined as “naturally derived bioactive compounds that are found in foods, dietary supplements and herbal products, and have health promoting, disease preventing, or medicinal properties”. The term, coming from the conjunction among nutrition and pharmaceutics, was coined in 1989 by Stephen De Felice [12].

Dietary phytochemicals belong to this group. They are non-nutrients compounds from a wide range of plant-derived foods. Despite the translational gap among basic and clinical research, the current understanding of the molecular interactions between phytochemicals and immune-inflammatory and oxidative stress pathways could help in designing effective nutritional strategies to delay ageing and age-related diseases. In particular, it has been claimed that dietary phytochemicals trigger a condition defined hormesis [1, 8, 13, 14]. This process is due to low-doses of bioactive compounds that act as mild stressors to induce adaptive expression of stress-protective genes and enhance resistance to mechanisms that determine ageing. The signal passes through the modulation of kinases, leading to the activation of downstream targets, among which FoxO3A and sirtuins. In this last case, it is to note the known effect of resveratrol, phytochemical contained in the grape, that, downstream, inhibits the NF-kB pathway, with interesting anti-inflammatory effects, although it is not totally clear and contrasting results exist [15]. *In vitro* and *in vivo* evidences suggested that many phytochemicals can affect the expression of numerous genes encoding pro-survival proteins, including antioxidant enzymes, neurotrophic or anti-apoptotic factors [1, 8].

Extra-virgin olive oil (EVOO) is full of these compounds, such as hydroxytyrosol, tyrosol and secoiridoids. It is extracted from olive fruits of *Olea Europea* and its health beneficial effects are well established. An extensive literature demonstrated that they can be attributed to many different substances belonging to the phenolic fraction of EVOO. However, the concentration of these molecules is strongly affected by belonging to a particular *cultivar*, by agronomic and environmental factors, and by the extraction and storage conditions [8, 16, 17].

Interestingly, experimental evidence suggests that hydroxytyrosol is not only effective in removing reactive oxygen species generated by impaired redox balance but it may also be a potent inducer of phase II detoxifying enzymes and an enhancer of mitochondrial biogenesis. Indeed, its effect is mostly observed at the transcriptional level through modulation of the redox-sensitive nuclear factor-E_2_-related factor 2 (Nrf2), which regulates gene expression of several phase II detoxifying enzymes and supports the structural and functional integrity of the mitochondria. For instance, in a study performed on rats, hydroxytyrosol supplementation improves neurogenesis and cognitive function through increased activity of the transcription factors FoxO1 and FoxO3, as well as Nrf2, resulting in decreased oxidative stress and increased mitochondrial function [18].

Several health claims for EVOO and its derivatives have been assessed in recent years but only one was authorized in Europe. It relates the impact of olive phenolic compounds on the protection of blood lipids from oxidative stress: “A daily intake of 20 g of olive oil, which contains at least 5 mg of hydroxytyrosol and its derivatives (e.g., oleuropein and tyrosol) provides the expected beneficial effects” [19].

Phytochemicals are also ingredients of many dietary supplements commercially available to prevent or ameliorate specific diseases, including age-related ones. The majority of these products are not substantiated by solid scientific evidences and have not been approved yet by the EFSA and/or FDA. Beyond them, functional foods are widely used, especially in Japan and in USA. Although a universal definition of them does not exist, they are often considered as foods with healthy properties that contain bioactive compounds naturally or added to obtain processed foods.

However, further observational studies and dietary intervention trials in large cohorts of healthy subjects are essential to evaluate whether these foods and compounds can help to prevent age-related disorders.

### The series

To discuss the potential relevance of nutrigerontology in the attainment of successful ageing and longevity, three scientific studies and two reviews have been assembled in this series.

As discussed by Passarino et al., on the whole, although the genetic factors account for only 25 % of human lifespan, the knowledge of the genetic basis of longevity may give significant hints on modulating lifestyle, in order to extend health span. That is, a few subjects can attain successful ageing and longevity thanks to a lucky combination of polymorphisms, which allow them to have an efficient metabolism or an efficient response to different types of stress. Most of the others can reach similar results by targeting the same pathways with appropriate lifestyle or interventions to slow ageing. In this context, the importance of epigenetic factors, both as biomarkers of ageing and target of interventions will certainly grow in the forthcoming studies [20].

The aim of the study of Accardi et al., was to analyse the nutraceutical properties of table green olives *Nocellara del Belice*, a traditional Mediterranean food, since little is known about the role of olives as nutraceutics. After the intake of 12 olives a day for 30 days, a significant decrease of malondialdehyde, a molecule related to oxidative stress, was observed. In addition, the level of interleukin-6 (IL-6) underwent a significant reduction, demonstrating how this food could be able to modulate the inflammatory response. Moreover, it was noteworthy the reduction of fat mass with an increase of muscle mass, suggesting a possible effect on long time assumption of table olives on body mass variation [9].

In Barera et al. study, the authors considered the nutraceutical effects of β-glucans, alimentary fibers, added to pasta, in order to produce a functional food. After 30 days of pasta intake, they obtained encouraging results with a significant decrease of low-density lipoprotein cholesterol, IL-6 and advanced glycation end-product levels. In fact, MedDiet is also characterized by a large intake of fibers, which contribute to lowering cholesterol and are positively associated with colon cancer prevention. However, in Mediterranean towns most people do not have a close adherence to MedDiet, hence adding fibers to pasta would achieve the same effects [21].

Data from dietary intervention studies of Carruba et al. underlined some interesting aspects related to mechanisms underpinning both biological and clinical effects of nutrition and specific activities of Mediterranean food components. In particular, the authors provided evidence that MedDiet may regulate oestrogen metabolism in postmenopausal women. In fact, it seems that the formation of potentially harmful genotoxic oestrogen compounds is remarkably reduced by the adoption of a traditional Mediterranean dietary model, while the levels of parent hormone estradiol become slightly increased. So, this result would imply that traditional Mediterranean food reduce the risk of developing breast cancer, while limiting the side effects of oestrogen withdrawal in menopause. Technological innovation and prototypical industrialization of either, processes or products, could be used to obtain traditional Mediterranean food with high health potential and market capacities. Precisely, the production of monocultivar EVOOs revealed that they may have differential activity on cellular and metabolic processes, eventually leading to produce highly characterized EVOOs with a preferential use for the prevention and care of various chronic diseases [22].

The review of Davinelli et al., attempted to summarise recent evidences about phytochemicals as anti-oxidants and anti-inflammatories. In fact, as previous mentioned, they may act as positive modulators of inflammation and oxidation by attenuating pro-inflammatory signalling associated with the redox imbalance that occurs in brain ageing. They also discussed the need to initiate long-term nutritional intervention studies in healthy subjects. In fact, their manuscript highlighted crucial aspects but that require further studies to determine the effective physiological concentrations and to explore the real impact of dietary phytochemicals in preserving brain health before the onset of symptoms leading to cognitive decline and inflammatory neurodegeneration [23].

## Discussion

As stated by Kolovou et al. [3] during the last two centuries the average lifespan has increased at a rate of approximately 3 months/year in both sexes. The most important steps in prolonging human lifespan were the decrease of child and maternal deaths, the lowering of infant and juvenile mortality rate due to, respectively, vaccination and treatment of infectious diseases. Moreover, in the last decades, the survival of elderly people improved thanks to secondary and primary prevention of ageing-related diseases and, particularly, coronary heart one [3].

So, all the factors above mentioned contribute to the fact that oldest old people are becoming the population with the fastest growth in Western World. Although the average life expectancy is increasing dramatically, the healthy lifespan is not going at the same pace. Hence, ageing and age-associated diseases are emerging as among the greatest challenges and financial burdens, faced by developed and developing countries [7].

Data from experimental studies in model organisms have consistently shown that both chronic dietary restriction, affecting nutrient signalling pathways, and mutations in nutrient and growth signalling pathways can extend longevity by 30–50 % [6, 7]. Also, they can lower the prevalence of age-related loss of function, including immunosenescence and multiple diseases, such as cancer, cardiovascular disease, and neurodegeneration [6, 7, 24].

In addition, several experimental data clearly demonstrate the hormetic effects of phytochemicals by inducing cellular stress resistance mechanisms [1, 8]. In designing human intervention studies to provide high-quality evidence for their health benefits, the following factors have to be considered: 1) the phytochemicals need to be sufficiently characterised, as well as its optimal physiologic dose, 2) the characteristics of targeted populations including their nutritional status, health condition, and genetic background have to be taken into account, 3) clinically relevant, sensitive, reproducible, and feasible endpoints have to be identified, 4) length of the intervention have to be commonly agreed [24–26]. However, even though experimental data have not always translated to a definitive clinical effect, the antioxidant and anti-inflammatory properties of phytochemicals have been widely accepted.

## Conclusion

The new findings presented in the experimental studies of the series give a great achievement for the food and farming industry, especially in Sicily, where local products represent a great potential resource. No approved healthy property and claim exist for them. Therefore, adding such products to the class of “healthy food” could represent a big deal. In the era of many expansive and mysterious longevity elixirs, they could represent a traditional, cheap and accessible “healthy food” to everyone. But more than for the single food, the Accardi’s study [9] highlights the importance to analyse local products that are traditional and easy to find. So, in Italy one should find phytochemicals in table green olives, in China in the Goji berries. In fact, the key is not a specific molecule in a specific food but its beneficial effect and the possibility for everyone to benefit.

The interesting effects of nutraceuticals and functional foods could represent a prevention for many age-related diseases, and, if not a total solution, at least a piece of their puzzle.

So, the possibility to create a dietary pattern, based on the combined strategy of the use of both nutraceuticals and functional foods, should permit to create a new therapeutic strategy based not only on a specific bioactive molecule or on a specific food but on a integrated approach that, starting from the local dietary habits, can be led to a “nutrafunctional diet” applicable worldwide.

Additional longitudinal observations on community-based cohorts are needed to confirm these data and investigate the biological mechanisms, including epigenetic ones, through which effects are induced, and to fully explore their therapeutic potential.

Nevertheless, nutrigerontology, putting together branches strictly related to ageing process, as biogerontology, medicine and nutrition, should be the key for achieving successful ageing and longevity.
